# New Strategies in the Cultivation of Olive Trees and Repercussions on the Nutritional Value of the Extra Virgin Olive Oil

**DOI:** 10.3390/molecules25102345

**Published:** 2020-05-18

**Authors:** Irene Dini, Giulia Graziani, Anna Gaspari, Francesca Luisa Fedele, Andrea Sicari, Francesco Vinale, Pierpaolo Cavallo, Matteo Lorito, Alberto Ritieni

**Affiliations:** 1 Department of Pharmacy, University of Naples Federico II, Via Domenico Montesano 49, 80141 Napoli, Italy; anna.gaspari@unina.it (A.G.); alberto.ritieni@unina.it (A.R.); 2 LINFA SCARL. Via Zona Industriale Porto San Salvo, 89900 Vibo Valentia, Italy; ricerca@laboratoriolinfa.it (F.L.F.); andrea@laboratoriolinfa.it (A.S.); 3 Department of Veterinary Medicine and Animal Productions, University of Naples Federico II, Via Federico Delpino 1, 80137 Napoli, Italy; francesco.vinale@unina.it; 4 Institute for Sustainable Plant Protection, National Research Council, Via Università 133, 80055 Portici (NA), Italy; matteo.lorito@unina.it; 5 Department of Physics, University of Salerno, Via Giovanni Paolo II, 132, 84084 Fisciano (SA), Italy; pcavallo@unisa.it; 6 Istituto Sistemi Complessi del Consiglio Nazionale delle Ricerche (ISC-CNR), 00185 Rome, Italy; 7 Department of Agricultural Science, University of Naples Federico II, Via Università 100, 80055 Portici, Italy

**Keywords:** *Trichoderma* spp., EVOO, secondary metabolites, phenolic identification, phenolic content, HRMS-Orbitrap, antioxidant activity

## Abstract

The health advantages of extra-virgin olive oil (EVOO) are ascribed mainly to the antioxidant ability of the phenolic compounds. Secoiridoids, hydroxytyrosol, tyrosol, phenolic acid, and flavones, are the main nutraceutical substances of EVOO. Applications of beneficial microbes and/or their metabolites impact the plant metabolome. In this study the effects of application of selected *Trichoderma* strains or their effectors (secondary metabolites) on the phenolic compounds content and antioxidant potential of the EVOOs have been evaluated. For this purpose, *Trichoderma virens* (strain GV41) and *Trichoderma harzianum* (strain T22), well-known biocontrol agents, and two their metabolites harzianic acid (HA) and 6-pentyl-α-pyrone (6PP) were been used to treat plants of *Olea europaea var.* Leccino and *var.* Carolea. Then the nutraceutical potential of EVOO was evaluated. Total phenolic content was estimated by Folin–Ciocalteau’s assay, metabolic profile by High-Resolution Mass spectroscopy (HRMS-Orbitrap), and antioxidant activity by DPPH and ABTS assays. Our results showed that in the cultivation of the olive tree, T22 and its metabolites improve the nutraceutical value of the EVOOs modulating the phenolic profile and improving antioxidants activity.

## 1. Introduction

The health benefits of the extra-virgin olive oil are ascribed mainly to phenolic compounds, among which the most concentrated are lignans (pinoresinol, acetoxypinoresinol, hydroxypinoresinol, etc.) and secoiridoids (ligstroside, oleuropein, etc.), with the latter located only in the Oleaceae family [1]. Other phenolics in EVOO are flavonoids (luteolin, apigenin, etc.), phenolic alcohols (tyrosol, hydroxytyrosol, etc.), and phenolic acids (hydroxybenzoic acid, ferulic acid, etc.) [2]. These substances modulate aging-associated processes and have antitumor, antiviral, anti-atherogenic, anti-inflammatory, antihepatotoxic, hypoglycemic, immunomodulatory [3,4,5,6], and anti-autoimmune (i.e., rheumatoid arthritis) properties [7]. The quality and content of the phenolic compounds in EVOO vary significantly according to the olive cultivar, environmental factors (altitude, agricultural practices, and amount of irrigation), oil extraction conditions (heating, added water, malaxation, pressure, centrifugation systems) and storage conditions [8]. The superior quality of the extra virgin olive oil is linked to olive fruits free of damage caused by pests, so pesticide (insecticides, fungicides, and herbicides) applications are used to enhance the number and size of olives. Unfortunately, residues from pesticides can pass into EVOO and determine health risks. Therefore, analytical procedures are carried out to determine their dosage in the oil. Organic agriculture is an alternative to the use of pesticides. These environmental systems depend on ecosystem management. Organic products are considered healthier and safer than conventional; therefore, they are required by the consumers despite their higher costs than conventional products. In the EU, subsidies were done to producers for compensating the lower incomes when they convert in organic the traditional cultivation (EC 2017) [9]. Biological control is one of the most appreciated alternatives against plant pathogens in a sustainable, environment-friendly strategy. The use of beneficial microbes have the benefit of the rhizosphere competence, allowing rapid establishment within the rhizosphere of a stable microbial community; suppression of pathogens by using a variety of mechanisms; overall improvement of plant health; plant growth promotion; enhancement of the nutrient availability and uptake, induction of host resistance similar to that stimulated by beneficial rhizobacteria, and positive change of the plant metabolome [10,11]. *Trichoderma* species are free-living fungi able to interact in the root, soil, and foliar environments with potential as biopesticides and biofertilizers [11,12]. They restore the beneficial balance of natural ecosystems by competing against the phytopathogens agents for space and nutrients [13]. They stimulate, in the plant, the production of secondary metabolites, including phenolic compounds, with health properties [14]. The commercial success of products containing these fungal antagonists can be attributed to the large volume of viable propagules that can be produced rapidly in several fermentation systems [15]. Biological diversity of the *Trichoderma* species produces a broad range of secondary metabolites; whose production varies according to the strain used [13]. Some of these biomolecules shown promising antifungal activity [16]. One factor that contributes to *Trichoderma* activities is related to the wide variety of metabolites that they produce. These metabolites have been found not only to directly inhibit the growth of pathogens, but also to increase disease resistance, enhance plant growth and modified plant metabolome [17]. Previous studies have shown that the *Trichoderma* T22 enhances the concentration of phenolics in the *Vitis vinifera* fruit and increases the tolerance of tomato plant to biotic and abiotic stresses by scavenging reactive oxygen species (ROS) and reutilizing oxidized glutathione and ascorbate [18,19]. It has been demonstrated that the *Trichoderma* GV41 improves antioxidant activity, total phenols concentration and profile in lettuce [14]. However, to date, nothing is known about the effects of these strains of *Trichoderma* on qualitative and quantitative profiles of the phenolic compounds in extra virgin olive oil. Therefore, the main objective of this study was to determine the possible impact of *Trichoderma* (T22 and GV41 on the nutraceutical value of the extra virgin olive oil produced by two *Olea europaea* varieties (Leccino and Carolea). Unfortunately, the use of the living fungus in agriculture is limited by the capacity of some strains to colonize every type of soils and plant roots, the impossibility of having directly proportional dose-response effects, and difficult storage conditions [20]. A solution to these problems is given using secondary metabolites of the *Trichoderma.* In this study, the ability of the tetrameric acid derivative with iron-binding activity (harzianic acid) and the food-grade volatile 6PP (pyrone 6-pentyl-α-pyrone) of improving the nutraceutical quality of the EVOO was also evaluated.

## 2. Results

### 2.1. Analyses of Phenolics

Q Exactive Orbitrap LC-MS/MS method allowed the identification and the quantification of the phenolic compounds in the samples. The identification of the phenolic compounds was made by comparing the retention times (Rt) to the mass spectra of the purified compounds and the standards. The ligstroside not commercially available was quantified by using the oleuropein in place of the authentic standard. Table 1 shows the retention time (RT) of phenolics obtained by UPLC-MS/MS.

A new chromatographic method was applied for the quantification of individual secondary metabolites, whose validation parameters were reported in Table 2. Limits of detection (LODs) range was from 0.02 to 1.0 mg/L, Limits of quantification (LOQs) 0.033 to 3.0 mg/L, and the linearity range between 88.7 and 1%.

Secoiridoids derivatives were the most representative phenolics in the two EVOO samples, and between these, the oleuropein-aglycone monoaldehyde, ligstroside-aglycone monoaldehyde, oleocanthal, and oleacein were the most abundant (Table 3).

The second group of phenolic compounds by concentration were phenolic alcohols (Table 4).

The third most abundant class of phenolic compounds were lignans (Table 5) followed by flavonoids (Table 6) and phenolic acids (Table 7).

Noteworthy was the content of luteolin in Carolea_oil_ and the ability of Ha biostimulation to improve it.

### 2.2. Phenol Content and Antioxidant Activity

**Figure 1:**
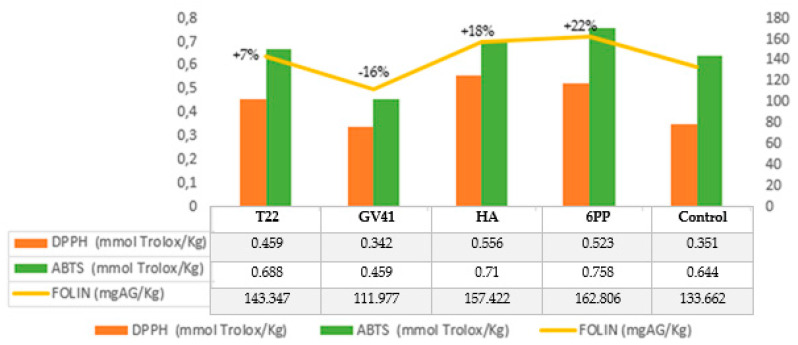
Phenolic content and antioxidant activity in the EVOO obtained by *Carolea* olives.

As shown in Figure 1, the EVOO obtained from Leccino variety olives had the highest content of phenols (EVOO*_Leccino_*: 133.662 mg/kg^−1^; EVOO*_Carolea_*: 77.871 mg·kg^−1^). The treatment of the Carolea olive trees with the biocontrol agent 6PP, compared to the untreated trees, improved the concentration of phenols in EVOO (+22%), followed by HA (+18%), and T22 (+7%), only the treatment with the living fungus GV41 decreased their concentration (−16%) (Figure 1).

**Figure 2:**
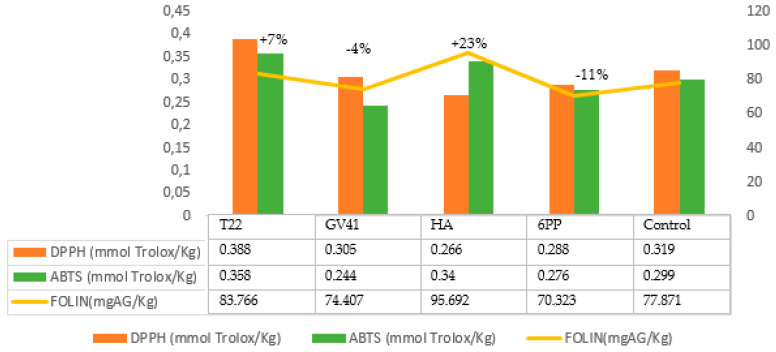
Phenolic content and antioxidant activity in the EVOO obtained by *Leccino* olives.

In Leccino EVOO, the highest concentration of phenols occurred in the oil produced from olives obtained by treating trees with HA (+23%), followed by T22 (+7%). On the contrary, the treatment of the olive trees with GV41 and 6PP decreased their concentration: −4% and −11%, respectively. (Figure 2).

DPPH and ABTS assays were used to determine the antioxidant activity of samples. A positive correlation was found between phenolic concentration and antioxidant activity measured by the ABTS test in all oil samples (Leccino_oil_ = 0.970322; *Cororea*_oil_ = 0.757275). Concerning the correlation between phenolic concentration and antioxidant activity measured by DPPH test, it was positive (0.91454) in Leccino_oil_ and negative in Corolea_oil_ (−0.09952).

### 2.3. Secoiridoids and Phenolic Alcohols Correlation

**Figure 3:**
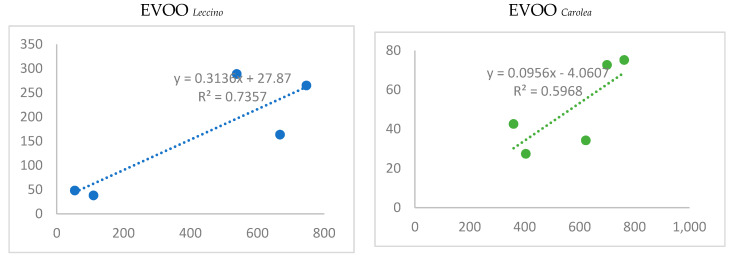
Correlation graphs of secoiridoids and phenolic alcohols.

Significant correlation indexes correlated secoiridoids and phenolic alcohols (EVOO *_Leccino_* − 0.7 and EVOO *_Carolea_*+ 0.6). These indexes were obtained by correlating the sum of the concentrations of the four most representative secoiridoids (oleuropein-aglycone monoaldehyde+ ligstroside-aglycone monoaldehyde + oleocanthal + oleacein) with the sum of the two phenolic acids (tyrosol + OHTyrosol) (Figure 3). The “+“ sign indicated that only a small part of secoiridoids degraded.

### 2.4. Variation (%) of the Most Representative Phenolics

**Figure 4:**
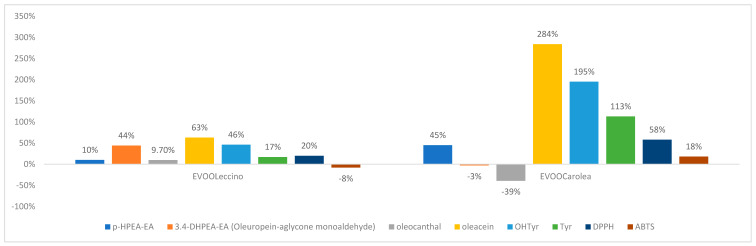
Variation (%) of the most representative secoiridoids, their degradation products, and antioxidant activity in EVOOs obtained from olive tree treated with T22 strains.

T22 strain determined in both oil samples an increase of the oleuropein-aglycon mono aldehyde of ≅45%. In the EVOO *_Carolea_*, was shown a remarkable negative variation (%) of ligstroside decarboxymethyl-aglycone (Figure 4).

**Figure 5:**
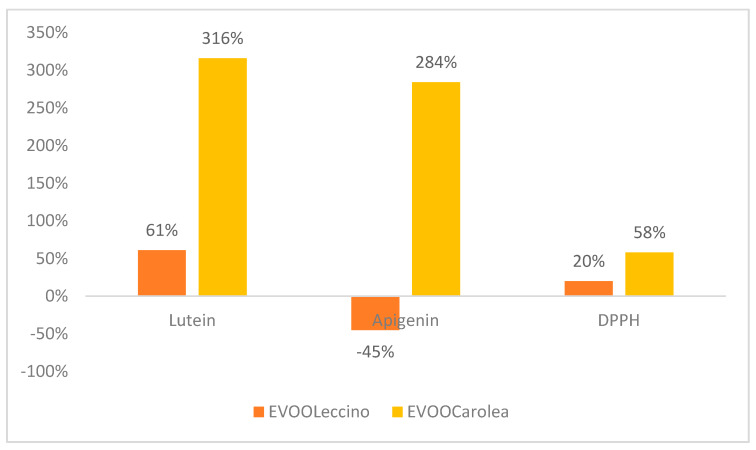
Variation (%) of flavonoids and antioxidant activity measured by DPPH test in EVOOs obtained from olive tree treated with HA.

HA treatment influenced flavonoid production in both monovarietal oils. The antioxidant activity of EVOO_Carolea_ was higher than EVOO _Leccino_ (Figure 5).

**Figure 6:**
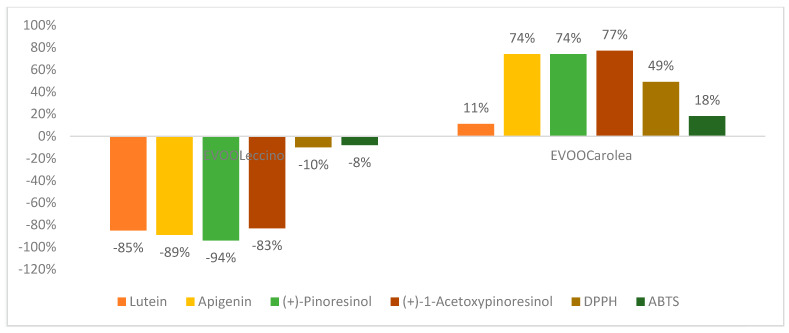
Variation (%) of flavonoids, lignans and antioxidant activity in the EVOO samples obtained from olive tree treated with 6PP.

The 6PP biostimulation interfered with the production of the flavonoids and the lignans in the olives (Figure 6).

**Figure 7:**
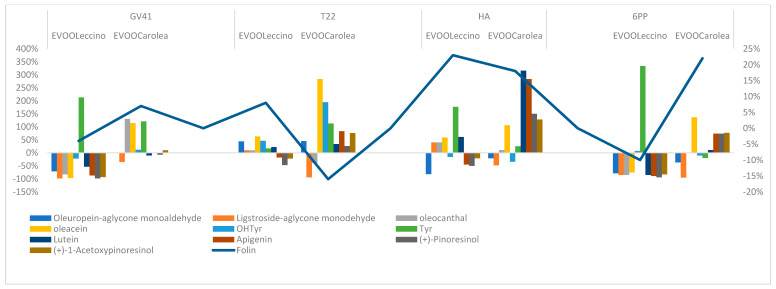
Variation (%) of the phenolic class of compounds and total phenol content in the EVOO samples.

The total phenolic content was strongly affected by variations of the concentration of oleacein, tyrosol and apigenin (Figure 7).

## 3. Discussion

**Figure 8:**
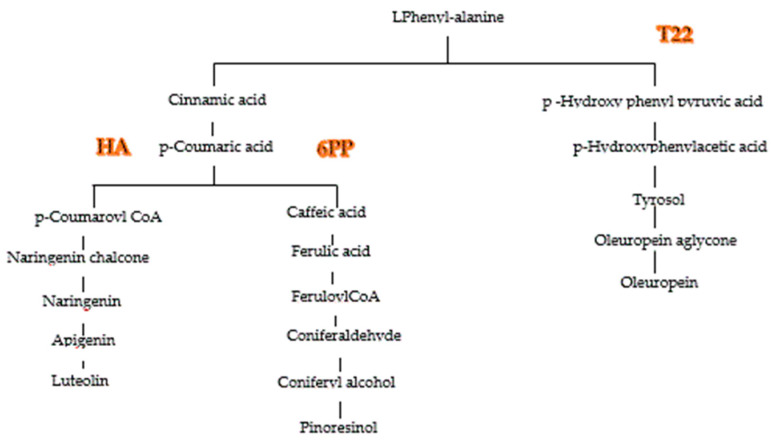
Interferences of *Trichoderma* T22 and its metabolites (HA and 6PP) on Shikimate pathway [23,30,31].

Two monovarietal EVVOs olives were analyzed to determine the possible impact on their nutraceutical properties when biocontrol strategy was used in the fields. This goal was obtained by treating two monovarietal olive trees (*Olea europaea* var. Leccino and *Olea europaea* var Carolea) with two strains of *Trichoderma* (GV41 and T22), and their metabolites HA and 6PP and evaluating the total content of phenols in the oil, determining the single phenol quality and quantity and comparing these data with the antioxidant activity of the oils. A remarkable variability was found in phenolic composition between the two sets of monovarietal EVOOs analyzed. The phenolic identification was obtained by using an Orbitrap platform in MS and MS/MS levels, and phenolic quantification was performed by using a UPLC-MS technology. The quantification method was validated in terms of linearity, precision, and sensitivity. The correlation factor of the calibration curve ≅1 established the first one, LODs, and the LOQs confirmed method sensitivity, and the relative standard deviation (RSD) <10% validated the repeatability. Nine secoiridoids, two phenolic alcohols, two lignans, two flavonoids, and six phenolic acids were characterized comparing the mass spectra with standards, except the ligstroside whose identification occurred comparing mass data with literature data, [21] and the hydroxybenzoic acid isomers, for which the retention times and the mass spectra were used. In all olive oils, the secoiridoid derivatives were the most abundant phenols, followed by phenolic alcohols, flavonoids, and phenolic acids. The lignans and the flavonoids were in the aglycon form since they degrade during the malaxation process. A significant correlation index between secoiridoids and phenolic alcohols (Figure 3) confirmed that tyrosol and the OHtyrosol were degradation products of ligstroside and oleuropein [22]. All biostimulant treatments, increased the total polyphenol content in EVOOs, except GV41 and 6PP in the EVOO *_Leccino._* The DPPH test and the ABTS method tested the antioxidant activity. The DPPH detected the ability of an antioxidant to transfer one electron to reduce any compound. The ABTS method determined the aptitude of the antioxidant to quench free radicals by hydrogen donation [23]. In this study, a significative correlation was found between the total phenolic content, and both tests used to determine the antioxidant activity. The DPPH measures were higher than that obtained with the ABTS test in the samples containing higher concentrations of flavonoids, *O*-diphenols and secoiridoids since DPPH test overestimates slow reacting antioxidants with many phenol groups as lutein, OHTyrosol and secoiridoid derivatives, able to donate hydrogen and improve radical stability by forming an intramolecular hydrogen bond between the free hydrogen of phenoxyl radicals, therefore the ABTS method is the best for the determination of the antioxidant activity in the oil [24]. The T22 biostimulation interferes above all with the production of secoiridoids in the olive. In both oil samples were found an increase of the oleuropein-aglycon mono aldehyde. A decreased concentration of ligstroside decarboxymethyl-aglycone was shown in the EVOO *_Carolea_*, probably due to transformation in its degradation product (tyrosol) (Figure 4). The biostimulation with the T22 strain enhanced the concentration of the total phenolic content of 7% in both oil samples (Figure 1 and Figure 2). This increase determines the growth of the antioxidant activity of ≅20% (DPPH = 22%; ABTS = 20%) in the EVOO *_Leccino,_* (Figure 2) and different measures of antioxidant activity in the EVOO *_Corolea_,* (Figure 1) according to the method used to determine it (DPPH test = 31% and ABTS test = 4%) (Figure 4), since the higher concentration of secoiridoids and flavonoids in EVOO *_Carolea_* than EVOO *_Leccino_*, were overestimated in the DPPH method. The biostimulation with HA increased the phenolic content (particularly the flavonoidic fraction) in both monovarietal oils (Figure 5). The higher concentration of flavonoids in EVOO *_Carolea_* than EVOO *_Leccino_* (Table 6), determined the overestimation in the DPPH method (Figure 5). The biostimulation with the 6PP metabolite decreased the total phenol concentration in the EVOO *_Leccino_* and enhanced it in the EVOO *_Corolea_* (Figure 1 and Figure 2). Consequently, the antioxidant activity decreased in the EVOO *_Leccino_* and improved in the EVOO *_Corolea_*. more than the control (Figure 6). The different variations of the polyphenol classes concentrations under the microbe or the microbe metabolites biostimulation (Figure 7) suggested that the nutraceutical properties [25] of the EVOO depended on the biostimulant used to grow olive trees (Figure 8). The biostimulation with T22 mainly enhanced the concentration of the secoiridoid fraction of phenols. As known, oleuropein is commercially available as a food supplement used to prevent the oxidation and the inflammatory damage, the cardiovascular and the cancer diseases, and as antiviral and antimicrobial agents [26]. Instead, the HA metabolite increased the flavonoids in the EVOOs. Luteolin has showed antitumorigenic, antimutagenic, antioxidative, immunomodulatory, and anti-inflammatory properties useful in cancer, cardiovascular diseases, and neurodegenerative pathologies prevention [27,28]. Finally, the 6PP metabolite improved lignans concentration in the EVOOs. Pinoresinol and acetoxypinoresinol intake has been related to LDL oxidation prevention, and health properties correlate to estrogen hormonal disfunction such as protection against cancer (prostate and breast) [29].

## 4. Materials and Methods

### 4.1. Study Area

This study was carried out in two experimental sites situated in Calabria, the most southern region of the Italian peninsula (ranges between 38°12′ and 40° latitude North and between 16°30′ and 17°15′ longitude East). The provinces of Calabria are: Catanzaro (CZ, regional capital), Reggio Calabria, Cosenza (CS), Crotone (KR), and Vibo Valentia (VV). The two experimental sites are in the villages of Cariati (CS), and Roccabernarda (KR). The climate of this Region is predominantly Mediterranean, temperatures are very mild, especially in the coastal plains. In the summer the heat is shared by the entire regional territory and only the altitude mitigates the heat or the breezes; peaks of over 35 °C are common. In the case of invasions of very hot African air, the temperatures exceed the 40 °C threshold. In Winter, on the other hand, temperatures remain mild with maxima greater than 10 °C on the coasts and cold in the internal areas and in the mountains, where the snow falls abundantly, and above 1000 m can persist throughout the period from December to March.

### 4.2. Plant Material

The possible impact of bioformulates was tested on two cultivars of *Olea europaea*: Leccino and Carolea. Plant material were given by Dr. Andrea Sicari (LINFA scarl, Vibo Valentia, Italy).

Plants (15 years old) in excellent nutritional and phytosanitary status with an adequate number of fruiting branches and a low ratio of wood and leaves were used for experimental purposes. The experimental field contained 20 plants split in 12 rows (3 rows per treatment). Six treatments were applied starting from February until July after plants sprouted. Each bioformulate (10^−6^ M HA, 10^−6^ M 6 PP, 10^6^ ufc/mL GV41, and 10^6^ ufc/mL T22) and one control sample (water treatment) were applied to the root system (drenching around the root system at 10 cm deep) and the leaves (10 L per row of which 5 L was spray and 5 L was drenching).

### 4.3. Fungal Material

Biological Control laboratories of the University of Naples Federico II provide the *Trichoderma* strains. The strains *Trichoderma harzianum* (T22 and GV41), and *Trichoderma harzianum* (M10 *Trichoderma harzianum* Rifai, anamorph ATCC^®^ 20847™, (LGC Standards S.r.l., Sesto San Giovanni, Mi Italy) were maintained on potato 125 dextrose agar (HiMedia, Laboratories Mumbai, India) and shielded with sterilized mineral oil (Sigma Aldrich, St. Louis, MO, USA).

### 4.4. Isolation and Characterization of Harzianic Acid

The *Trichoderma* strains M10 was used to produce the bioactive molecules. Mycelia were inoculated into 1L of sterile potato dextrose broth (PDB, HiMedia Mumbai, India). Cultures of each strain were grown for 30 days at 25 °C, and then vacuum-filtered through filter paper (Whatman No. 4, Brentford, UK). Ethyl acetate (EtOAc) was used to extract the filtrate (2 L). Organic fractions were dried with Na_2_SO_4_ and the solvent evaporated in vacuum at 35 °C. The red residue obtained from M10 was dissolved in CHCl_3_ and extracted three-times with NaOH 2M. Harzianic acid (HA) then precipitated with HCl 2M. The solid was recovered (135mg), solubilized and subjected to RP-18 vacuum chromatography (20 g Si gel RP-18, 40–63μm Sigma Aldrich, St. Louis, MO), eluting with a gradient of methanol (MeOH): H_2_O:CH_3_CN (0.5:9:0.5/*v*:*v*:*v* to 10:0:0/*v*:*v*:*v*). After separation, approximately 45mg of pure HA was collected. The compounds were detected spectrophotometrically on TLC (UV: λ 254 or λ 366 nm) and by dipping the plates in a 5% (*w*/*v*) ethanol solution of 2M H_2_SO_4_ and heating at 110 °C for 10min. The purified metabolites were characterized by NMR (Bruker AM 400 spectrometer; Bruker, Billerica, Massachusetts, USA) operating at 400 (1H) MHz using residual and deuterated solvent peaks as a reference standard or by LC-MS/MS QTOF (Agilent Technologies, Santa Clara, CA, USA) with a dual ESI (Electrospray Ionization) source, coupled to a DAD (Diode-Array Detection; Agilent Technologies, Santa Clara, CA, USA).

### 4.5. Oil Production

The oils samples were cold produced at a semi-industrial scale in a local two-phase mill. It was kept at a constant temperature (10 ± 2 °C) in dark bottles without headspace until analysis.

#### 4.5.1. Chemicals

All the chemicals used are from Sigma Aldrich St. Louis, MO, USA, unless specified differently.

#### 4.5.2. Extraction of Phenolic Compounds from Olive Oil

The method proposed by Vasquez Roncero [32] was used. In 25 mL hexane were put 25 g oil. 15 mL methanol:water (3:2 *v/v*) extracted the polar in three times. The extracts combined were treated once with 25 mL hexane. The solvent was evaporated in a rotary evaporator (Büchi, Switzerland) at 40° C. The insoluble residue was abundantly washed with CH_3_OH and filtered through 0.2 μm nylon filter and immediately stored at −18 °C until analysis.

#### 4.5.3. Ultra High Pressure Liquid Chromatograph

Polyphenol compounds were isolated and quantified by an Ultra High Pressure Liquid Chromatograph (UHPLC, Thermo Fisher Scientific, Waltham, MA, USA) equipped with a Dionex Ultimate 3000 degassing system, a quaternary UHPLC pump (Thermo Fisher Scientific, Waltham, MA, USA) working at 1250 bar, and a column (Thermo Scientific, Waltham, MA, USA) Accucore aQ 2.6 µm (100 × 2.1 mm) in a thermostated compartment (T = 30 °C). 5 µL of the sample was injected. The eluent phase consists of a gradient programmed as follows: 0 to 5 min −5% of phase B, 25 min −40% of phase B, 25.1 min −100% of phase B, 27 min −100% of phase B, 27.1 min −5% of phase B, 35 min −5% of phase B, 31 min −0% of phase B where phase A was H_2_O 0.1% of acetic acid and phase B was acetonitrile. The flow rate was 0.4 mL/min.

### 4.6. Mass Spectrometry Analysis

A Q Exactive Orbitrap LC-MS/MS (Thermo Fisher Scientific, Waltham, MA, USA) was used for experimental purposes provided of an ESI source (HESI II, Thermo Fisher Scientific, Waltham, MA, USA) [spray voltage −3.0 kV, capillary temperature 200 °C, auxiliary gas (N_2_ > 95%) ^15^, sheath gas (N_2_ > 95%) ^30^, auxiliary gas heater temperature 305 °C and S-lens RF level 50]. The mass detection was obtained in two acquisition modes: negative-ion modes (full scan; mass resolving power 35,000 full width at half maximum (at *m*/*z* 200), the automatic gain control target 1 × 105 ions for a maximum injection time of 200 ms, and scan range 100–1500 *m*/*z*, scan rate 2 s^−1^) and targeted selected ion monitoring [15 s-time window, quadrupole isolation window 1.2 *m*/*z*, and resolution power 35,000 full width at half maximum (at *m*/*z* 200)].

### 4.7. Validation of the Method Used to Quantify Single Phenols

Method was validated following AOAC instructions (AOAC 2012) [33]. The parameters analyzed were linearity, LOD, LOQ, repeatability, and reproducibility. Three points (in triplicate) were used to build the calibration curves of each compound. Method linearity was obtained from the regression coefficient of the calibration curve LOD (Limits of detection) and LOQ limits of quantification were calculated from the regression curve. Nine different concentrations of each phenolic standard three times gave intraday repeatability.

(1) $$\mathrm{LODs}=3\;\times \;\frac{\mathrm{standard}\;\mathrm{deviation}}{angular\;coefficient}\;;\;\mathrm{LOQs}=10\;\times \;\frac{\mathrm{standard}\;\mathrm{deviation}}{angular\;coefficient}$$

### 4.8. Total Phenolic Compounds

The total polyphenols amount was evaluated by using the Folin–Ciocalteau’s assay as reported by Singleton and Rossi (1965) [34]. In a falcon (15 mL), 2.5 mL dd H_2_O and 625 µL methanolic extract, 625 µL of Folin–Ciocalteau’s phenol reagent were shaken. After 6 min, 6.25 mL of 7% Na2CO3 solution was added to the mixture. The solution was diluted with 5 mL dd H_2_O and mixed. The absorbance (Lambda 25, PerkinElmer, Italy) of reagent blank was determined at 760 nm by spectrophotometer after incubation for 90 min at room temperature. All biological replicates of samples were analyzed in triplicate. Total phenolic content was expressed as mg gallic equivalents (GAE)/kg FW.

### 4.9. Antioxidant Activity Measurements

DPPH method. 2,2-Diphenyl-1-picrylhydrazyl (DPPH) radical-scavenging capacity was measured using the method described by Brand-Williams et al. (1995) [35]. Fraction aliquots (20 μL) were added to 3 mL of DPPH solution (6 × 10^−5^ mol/L) and the absorbance was determined at λ517 nm every 5 min until the steady state (Lambda 25, PerkinElmer, Italy). Calibration curve was obtained using 6-Hydroxy-2,5,7,8-tetramethylchroman-2-carboxylic acid (Trolox), a water-soluble analog of α-tocopherol, as standard and results were expressed as mmol Trolox equivalent (TE) kg^−1^ FW. All biological replicates of samples were analyzed in triplicate.

ABTS method. 2,2′-azinobis (3-Ethylbenzothiazoline-6-sulfonic acid) (ABTS) procedure modified from Re et al. was used (1999) [36]. A concentrate solution of the reagent (stock solution) was prepared dissolving 9.6 mg of ABTS in 2.5 mL of water and adding 44 mL of a solution made by dissolving 37.5 mg of potassium persulphate, K_2_S_2_O_8_, in 1 mL of water. The stock solution was kept in the dark at 4 °C for 8 h before use; the work solution was obtained from the stock solution by dilution using a 1:88 (*v/v*) ratio. Dilution was adjusted depending on the measured absorbance at λ734 nm (A734) in the work solution, until a value between 0.7 and 0.8. Subsequently, 100 μL of sample and 1 mL of work solution were added, and A734 was measured exactly after 2 min and 30 s. (Lambda 25, PerkinElmer, Italy). Calibration curve was obtained using 6-Hydroxy-2,5,7,8-tetramethylchroman-2-carboxylic acid (Trolox), a water-soluble analog of α- tocopherol, as standard and results were expressed as mmol Trolox equivalent (TE) kg^−1^ FW. All biological replicates of samples were analyzed in triplicate.

## 5. Conclusions

Our results confirmed the ability of the *Trichoderma harzianum* (strain T22) and its metabolites (6PP and HA) in the defense system of the *Olea europaea* tree and demonstrate that the ABTS test is the preferred method for determining the antioxidant activity in the EVOO. To the best of our knowledge, the present work is the first report that correlates the nutraceutical properties of the EVOO to a specific biostimulation method used in the field. Our results suggest new possibilities of using *Thricoderma* and its metabolites to select the nutraceutical properties of the EVOO and recommend the use of *Thricoderma* metabolites in olive tree cultivation to avoid some of the limitations related to the application of living microbes.

## Figures and Tables

**Table 1:** Analytical parameters of phenolics identification; all compounds were monitored in negative mode.

Phenolic Compounds	RT (min)	Formula	Theoretical *m*/*z* of Deprotonated Molecular Ions [M − H]^−^	Experimental *m*/*z* of Deprotonated Molecular ions [M − H]^−^	Calculated Errors ∆ppm	Fragments	Collision Energy (eV)
***Phenolic acids***							
Vanillic acid	4.30	C_8_H_8_O_4_	167.03498	167.03522	1.44	152.01143	20
*p*-Coumaric acid	9.71	C_9_H_10_O_5_	163.04007	163.04028	1.29	119.05023	20
Cinnamic acid	11.54	C_9_H_8_O_2_	147.04515	147.04536	1.43	103.04501	20
Ferulic acid	11.81	C_10_H_10_O_4_	193.05063	193.05084	1.09	178.02685	20
4-Hydroxybenzoic acid	2.57	C_7_H_6_O_3_	137.02442	137.02456	1.02	93.03431	12
3-Hydroxybenzoic acid	2.88	C_7_H_6_O_3_	137.02442	137.02458	1.17	93.03431	12
**Flavonoids**							
Apigenin	19.12	C_15_H_10_O_5_	269.04555	269.04597	1.56	225.05592	35
Luteolin	19.07	C_15_H_10_O_6_	285.04046	285.04106	2.10	133.02940	30
**Lignans**							
(+) Pinoresinol	17.00	C_20_H_22_O_6_	357.13436	357.13487	1.43	151.03961	40
(+) 1-Acetoxypinoresinol	19.10	C_22_H_24_O_8_	415.13984	415.14007	0.55	415.13821	40
**Phenolic Alcohols**							
Hydroxytyrosol (3,4 DHPEA)	1.60	C_8_H_10_O_3_	153.05572	153.05580	0.52	123.04561	12
Tyrosol (*p*-HPEA)	2.75	C_8_H_10_O_2_	137.06080	137.06096	1.17	119.05022	12
**Secoiridoids**							
Elenolic acid	13.14	C_11_H_14_O_6_	241.07176	241.07212	1.49	209.04573	10
Oleacein (3.4 DHPEA-EDA)	16.14	C_17_H_20_O_6_	319.11871	319.11898	0.85	301.1082	15
Oleuropein	16.69	C_25_H_32_O_13_	539.17701	539.17767	1.22	377.12393	20
Ligstroside	18.25	C_25_H_32_O_12_	523.18210	523.18279	1.32	361.12914	12
Ligstroside-decarboxymethyl aglycone oleocanthal(*p*-HPEA-EDA)	18.59	C_17_H_20_O_5_	303.12380	303.12441	2.01	301.1082	12
Secologanoside	19.49	C_16_H_21_O_11_	389.1092	389.109258	0.59	345.1195	12
Oleuropein-aglycone mono-aldehyde (3.4 DHPEA-EA)	21.25	C_19_H_22_O_8_	377.12419	377.12442	0.61	345.09790	12
*p*-HPEA-EA (Ligstroside- aglycone monoaldehyde)	21.59	C_19_H_22_0_7_	361.12145	361.12141	−0.11	291.1122	21

**Table 2:** Validation parameters of the quantification method.

Phenolic Compounds	Linearity (mg/L)	R^2^	LOD (mg/L)	LOQ (mg/L)	Intraday RSD % (*n* = 3), 50 mg/L
**Phenolic Acids**					
Vanillic acid	1–50	0.887	0.200	0.600	1.1
*p*-Coumaric acid	1−50	1.000	0.100	0.300	1.8
Cinnamic acid	1−50	0.991	0.200	0.600	0.9
4-Hydroxybenzoic acid	1−50	0.998	0.207	0.622	0.9
3-Hydroxybenzoic acid	1−50	0.995	0.205	0.622	1.1
**Flavonoids**					
Apigenin	0.5−50	0.899	0.066	0.200	2.1
Luteolin	0.5−50	0.991	0.066	0.200	1.4
**Lignans**					
(+) Pinoresinol	1−50	0.999	0.02	0.060	0.5
(+)1-Acetoxypinoresinol	1−50	0.899	0.233	0.700	1.5
**Phenolic Alcohols**					
3.4 DHPEA (Hydroxytyrosol)	1−50	0.992	0.666	2.000	3.0
*p*-HPEA (Tyrosol)	1−50	0.991	0.040	0.133	1.6
**Secoiridoids**					
Elenolic acid	1−50	0.991	0.333	1.000	0.7
Oleuropein	1−50	0.991	0.166	0.500	5.0
Ligstroside	1−50	0.991	0.166	0.500	4.0
Oleocanthal	1−50	0.899	0.416	1.250	3.0
Secologanoside	1−50	0.967	0.333	1.000	2.1
3.4-DHPEA-EA (Oleuropein-aglycone monoaldehyde)	1−50	0.998	1.000	3.000	2.1
p-HPEA-EA (Ligstroside- aglycone monoaldehyde)	1−50	0.999	0.033	0.100	0.7
3.4 DHPEA-EDA (Oleacein)	1−50	0.991	0.033	0.100	1.1

**Table 3:** Content of secoiridoids in EVOO (mg/kg).

	Oleuropein	Ligstroside	Secologanoside	Elenolic Acid	p-HPEA-EA	3.4-DHPEA-EA (Oleuropein-aglycone monoaldehyde)	p- HPEA-EDA (Ligstroside- decarboxymethyl aglycone)	3.4-DHPEA-EDA (oleacein)
***Leccino cultivar***								
T22	0.051 ± 0	0.016 ± 0.004	0.022 ± 0.003	0.782 ± 0.006	113.34 ± 0.234	151.672 ± 0.018	113.34 ± 0.234	368.416 ± 5.474
GV41	0.062 ± 0.002	0.002 ± 0.001	0.012 ± 0.001	0.176 ± 0.002	17.71 ± 0.019	30.307 ± 0.503	17.71 ± 0.019	6.207 ± 0.09
HA	0.053 ± 0.003	0.015 ± 0.002	0.083 ± 0.008	1.317 ± 0.023	144.889 ± 1.349	18.578 ± 2.467	144.889 ± 1.349	359.45 ± 2.078
6PP	0.053 ± 0.001	0.001 ± 0.001	0.004 ± 0.001	0.126 ± 0.002	15.661 ± 0.343	22.335 ± 0.41	15.661 ± 0.343	56.547±0.319
Control	0.046 ± 0.003	0.007 ± 0.001	0.01 ± 0	2.704 ± 0.144	103.342 ± 0.553	105.488 ± 0.506	103.342 ± 0.553	226.173 ± 0.065
***Carolea cultivar***								
T22	0.056 ± 0.002	0.012 ± 0.001	0.011 ± 0	2.511 ± 0.014	139.98 ± 1.635	305.157 ± 1.554	139.98 ± 1.635	114.526 ±0.321
GV41	0.064 ± 0.003	0.027 ± 0	0.118 ± 0.001	1.535 ± 0.004	249.437 ± 1.244	208.585 ± 3.183	149.437 ± 1.244	55.067 ± 0.1
HA	0.069 ± 0.001	0.023 ± 0.002	0.181 ± 0.005	5.555 ± 0.071	119.875 ± 0.849	166.79 ± 0.291	119.875 ± 0.849	52.865 ± 0.406
6PP	0.053 ± 0.001	0.001 ± 0.001	0.004 ± 0.001	2.226 ± 0.01	108.81 ± 1.891	133.9 ± 1.021	108.81 ± 1.891	60.665 ± 0.169
Control	0.377 ± 0.431	0.038 ± 0.006	0.124 ± 0.009	1.28 ± 0.237	108.172 ± 15.044	210.729 ± 6.933	228.172 ± 15.044	25.634 ± 6.69

**Table 4:** Content of phenolic alcohols in EVOO (mg/kg).

	3.4 DHPEA (Hydroxytyrosol)	*p*-HPEA (Tyrosol)
***Leccino cultivar***		
T22	0.928 ± 0.008	155.108 ± 0.731
GV41	0.498 ± 0.016	164.541 ± 0.932
HA	0.535 ± 0.01	146.029 ± 0.881
6PP	0.683 ± 0.005	228.288 ± 2.377
Control	0.636 ± 0.007	52.657 ± 0.562
***Carolea cultivar***		
T22	0.308 ± 0.007	72.356 ± 0.893
GV41	0.294 ± 0.014	74.904 ± 3.824
HA	0.174 ± 0.002	42.38 ± 0.75
6PP	0.238 ± 0.003	27.106 ± 0.901
Control	0.263 ± 0.009	33.916 ± 0.403

**Table 5:** Content of lignans in EVOO (mg/kg).

	(+)-Pinoresinol	(+)-1-Acetoxypinoresinol
***Leccino cultivar***		
T22	0.855 ± 0.024	38.972 ± 1.817
GV41	0.033 ± 0.001	3.655 ± 0.108
HA	0.799 ± 0.007	39.227 ± 0.554
6PP	0.104 ± 0.003	8.715 ± 0.321
Control	1.608 ± 0.01	49.807 ± 0.558
***Carolea cultivar***		
T22	0.376 ± 0.001	33.433 ± 0.882
GV41	0.276 ± 0.003	20.821 ± 0.511
HA	0.744 ± 0.02	43.291 ± 0.269
6PP	0.517 ± 0.005	33.547 ± 0.516
Control	0.298 ± 0.063	18.967 ± 0.461

**Table 6:** Content of flavonoids in EVOO (mg/kg).

	Luteolin	Apigenin
***Leccino cultivar***		
T22	0.634 ± 0.005	0.075 ± 0.001
GV41	0.244 ± 0.012	0.012 ± 0
HA	0.835 ± 0.009	0.086 ± 0.001
6PP	0.08 ± 0.002	0.01 ± 0
Control	0.52 ± 0.015	0.091 ± 0
***Carolea cultivar***		
T22	2.749 ± 0.009	0.188 ± 0.007
GV41	1.841 ± 0.004	0.102 ± 0.001
HA	8.505 ± 0.002	0.395 ± 0.005
6PP	2.261 ± 0.047	0.179 ± 0.003
Control	2.045 ± 0.346	0.103 ± 0.01

**Table 7:** Content of phenolic acids in EVOO (mg/kg).

	4-Hydroxy Benzoic Acid	3-Hydroxy Benzoic Acid	Vanillic Acid	p-Coumaric Acid	Cinnamic Acid	Ferulic Acid
*Leccino cultivar*						
T22	0.419 ± 0.013	0.031 ± 0.008	0.356 ± 0.03	0284 ± 0.002	0.143 ± 0.002	0.144 ± 0.004
GV41	0.027 ± 0.002	0.02 ± 0.002	0.08 ± 0.001	0.109 ± 0.005	0.023 ± 0.001	0.036 ± 0.001
HA	0.501 ± 0.013	0.055 ± 0.01	0.599 ± 0.011	0.373 ± 0.004	0.165 ± 0.003	0.179 ± 0.002
6PP	0.044 ± 0.001	0.007 ± 0	0.057 ± 0.001	0.036 ± 0.001	0.018 ± 0.001	0.019 ± 0
Control	0.56 ± 0.007	0.075 ± 0.004	1.23 ± 0.066	0.381 ± 0.007	0.176 ± 0.001	0.32 ± 0.005
*Carolea cultivar*						
T22	0.166 ± 0.001	0.218 ± 0.004	1.142 ± 0.006	1.23 ± 0.004	0.361 ± 0.014	0.38 ± 0.003
GV41	0.226 ± 0.003	0.139 ± 0.005	0.698 ± 0.002	0.824 ± 0.002	0.196 ± 0.001	0.294 ± 0.003
HA	0.345 ± 0.006	0.437 ± 0.008	2.527 ± 0.032	3.805 ± 0.001	0.759 ± 0.009	0.888 ± 0.011
6PP	0.25 ± 0.008	0.174 ± 0.002	1.013 ± 0.004	1.012 ± 0.021	0.344 ± 0.005	0.313 ± 0.001
Control	0.308 ± 0.045	0.119 ± 0.003	0.583 ± 0.108	0.915 ± 0.155	0.197 ± 0.019	0.307 ± 0.007
